# Early combination therapy of COVID-19 in high-risk patients

**DOI:** 10.1007/s15010-023-02125-5

**Published:** 2023-11-29

**Authors:** Hans Martin Orth, Charlotte Flasshove, Moritz Berger, Tessa Hattenhauer, Kaja D. Biederbick, Rebekka Mispelbaum, Uwe Klein, Jannik Stemler, Matthis Fisahn, Anna D. Doleschall, Ben-Niklas Baermann, Eva Koenigshausen, Olga Tselikmann, Alexander Killer, Clara de Angelis, Smaranda Gliga, Johannes Stegbauer, Nikolai Spuck, Gerda Silling, Jürgen K. Rockstroh, Christian P. Strassburg, Peter Brossart, Jens P. Panse, Björn-Erik Ole Jensen, Tom Luedde, Christoph Boesecke, Annkristin Heine, Oliver A. Cornely, Malte B. Monin

**Affiliations:** 1 Centre for Integrated Oncology (CIO), Aachen, Bonn, Cologne, Düsseldorf, (ABCD), Aachen, Germany; 2 Department of Gastroenterology, Hepatology and Infectious Diseases, Medical Faculty and University Hospital Düsseldorf, Heinrich-Heine-University Düsseldorf, Moorenstraße 5, 40225 Düsseldorf, Germany; 3 Institute for Medical Biometry, Informatics and Epidemiology, Bonn University Hospital, Venusberg-Campus 1, 53127 Bonn, Germany; 4 Department of Oncology, Hematology, Rheumatology and Immune-Oncology, University Hospital Bonn, Venusberg-Campus 1, 53127 Bonn, Germany; 5 Department I of Internal Medicine, European Diamond Excellence Centre for Medical Mycology (ECMM), University of Cologne, Faculty of Medicine, and University Hospital of Cologne, Kerpener Str. 62, 50937 Cologne, Germany; 6 Institute of Translational Research, Cologne Excellence Cluster On Cellular Stress Responses, University of Cologne, Kerpener Str. 62, 50937 Cologne, Germany; 7 German Centre for Infection Research (DZIF), Partner-Site Cologne-Bonn, Kerpener Str. 62, 50937 Cologne, Germany; 8 Department of Oncology, Hematology, Hemostaseology and Stem Cell Transplantation, University Hospital RWTH Aachen, Pauwelsstraße 30, 52074 Aachen, Germany; 9 Department of Hematology, Oncology, and Clinical Immunology, Medical Faculty and University Hospital Düsseldorf, Heinrich-Heine-University Düsseldorf, Moorenstraße 5, 40225 Düsseldorf, Germany; 10 Department of Nephrology, Medical Faculty and University Hospital Düsseldorf, Heinrich-Heine-University Düsseldorf, Moorenstraße 5, 40225 Düsseldorf, Germany; 11 Department of Internal Medicine I, University Hospital Bonn, Venusberg-Campus 1, 53127 Bonn, Germany; 12 German Centre for Infection Research (DZIF), Partner-Site Cologne-Bonn, Venusberg-Campus 1, 53127 Bonn, Germany; 13 Johanniter-Kliniken Bonn GmbH, Johanniter-Krankenhaus Bonn, Bonn, Germany

**Keywords:** COVID-19, Immunocompromised host, Dual anti-SARS-CoV-2 therapies, Prolonged viral shedding, Individualized therapeutic approaches

## Abstract

**Purpose:**

Prolonged shedding of severe acute respiratory syndrome coronavirus 2 (SARS-CoV-2) has been observed in immunocompromised hosts. Early monotherapy with direct-acting antivirals or monoclonal antibodies, as recommended by the international guidelines, does not prevent this with certainty. Dual therapies may therefore have a synergistic effect.

**Methods:**

This retrospective, multicentre study compared treatment strategies for corona virus disease-19 (COVID-19) with combinations of nirmatrelvir/ritonavir, remdesivir, molnupiravir, and/ or mABs during the Omicron surge. Co-primary endpoints were prolonged viral shedding (≥ 10^6^ copies/ml at day 21 after treatment initiation) and days with SARS-CoV-2 viral load ≥ 10^6^ copies/ml. Therapeutic strategies and risk groups were compared using odds ratios and Fisher’s tests or Kaplan−Meier analysis and long-rank tests. Multivariable regression analysis was performed.

**Results:**

144 patients were included with a median duration of SARS-CoV-2 viral load ≥ 10^6^ copies/ml of 8.0 days (IQR 6.0–15.3). Underlying haematological malignancies (HM) (*p* = 0.03) and treatment initiation later than five days after diagnosis (*p* < 0.01) were significantly associated with longer viral shedding. Prolonged viral shedding was observed in 14.6% (*n* = 21/144), particularly in patients with underlying HM (OR 3.5; 95% CI 1.2–9.9; *p* = 0.02). Clinical courses of COVID-19 were mild to moderate with only few adverse effects potentially related to combination treatment.

**Conclusion:**

Early combination treatment of COVID-19 effectively prevented prolonged viral shedding in 85.6% of cases. Considering the rapid viral clearance rates and low toxicity, individualized dual therapy approaches may be beneficial in high-risk patients.

## Introduction

Based on the interventional studies and real-world data, the World Health Organization (WHO) recommends early monotherapy for coronavirus disease 2019 (COVID-19) in patients at risk of severe courses [1–4]. Several direct-acting antivirals (DAAs) and monoclonal antibodies (mABs) are therefore available. Older age, immunodeficiency, the extent of immune response after vaccination and/or infection and the number of risk factors are associated with a more severe clinical course [5]. Moreover, despite early initiation of monotherapy in immunocompromised hosts, shedding of severe acute respiratory syndrome coronavirus 2 (SARS-CoV-2) was prolonged [6]. This favours the development of escape mutations against mAB therapies [7]. We form a network of four large university hospitals in North Rhine-Westphalia (Aachen, Bonn, Cologne and Düsseldorf) with over 5,000 beds and numerous highly specialised outpatient clinics. In this way, we provide COVID-19 care mainly for patients with haematological malignancies (HM) and for patients on immunosuppressive medication after solid-organ transplantation (SOT). These patients face prolonged infectiousness that limits their participation in daily life, including access to the medical system, which can interfere with appropriate management of underlying diseases [8].

Combination treatment to avoid the development of drug resistance or to improve therapeutic efficacy is well established, for example in human immunodeficiency virus infection or tuberculosis. The availability of three DAAs (nirmatrelvir/ritonavir, remdesivir, molnupiravir) and various mABs enables combination treatment against COVID-19. Molnupiravir-nirmatrelvir/ritonavir was more effective in vitro and in animal models, and improved survival in mice [9–11]. Molnupiravir−remdesivir combination was synergistic in hamsters [12]. In a case series of immunocompromised patients, the addition of a mAB to remdesivir was shown to increase the chance of sustained viral clearance as compared to remdesivir monotherapy [13]. Moreover, combination of nirmatrelvir/ritonavir with an mAB was effective in a small case series of immunocompromised patients with sustained viral shedding [14]. In humans, current experiences with combined DAAs are based on the few case reports and small case series [15].

Despite these encouraging reports, optimal combinations are unknown. In our large cohort of immunocompromised patients, we analysed first-line COVID-19 combination regimens for virological and clinical outcomes and safety.

## Patients and methods

This is a retrospective, multicentre, observational cohort study conducted during the Omicron surge at four German university hospitals forming the Centre for Integrated Oncology Aachen Bonn Cologne Düsseldorf (CIO ABCD). Cases of mainly immunocompromised patients who received first-line combination treatment with DAAs ± mABs against COVID-19 between March 2022 and April 2023 were analysed. Therapeutic decisions were at the discretion of the treating physicians. The study was performed in accordance with the Declaration of Helsinki. Owing to the retrospective study design, ethical approval and/or patient information was not required according to the North Rhine-Westphalian legislation. According to internal standards, however, we submitted lead ethics proposals, which were approved by the Ethics Committees of the Medical Faculty of the Heinrich-Heine University Düsseldorf, Germany (study no. 2022-2240) and of the Medical Faculty of the University Hospital Bonn, Germany (file number 469/22).

For inclusion, patients were at risk of severe courses of COVID-19 and received first-line combination therapies for COVID-19 containing at least two of nirmatrelvir/ritonavir (administered five days orally), remdesivir (administered three, five or ten days intravenously), molnupiravir (administered five days orally), and/ or one mAB (administered intravenously or intramuscularly as single shot) at standard doses. The effectiveness of the mABs was tested in each individual case [16]. The dose of the mAB was doubled in cases of restricted neutralization capacity and/or in cases, in which sequencing of the viral genome was not performed, ensuring effectiveness. Combination therapy was assumed if the individual substances were administered overlapping for at least one day. Diagnosis of SARS-CoV-2 infection was confirmed by real-time reverse-transcriptase polymerase chain reaction (RT-PCR) with or without viral sequencing. If viral sequencing was not available, the regionally predominant variant was assumed. The date of diagnosis was considered the date of first positive RT-PCR. Treatment initiation later than five days after diagnosis was defined as late treatment initiation.

Co-primary endpoints were days with SARS-CoV-2 viral load ≥ 10^6^ RNA copies/ml and occurrence of prolonged viral shedding, both overall, and compared among subgroups of antiviral therapy strategies (2 × DAAs ± 1 × mAB versus 1 × DAA plus 1 × mAB). Prolonged viral shedding was defined as SARS-CoV-2 viral load ≥ 10^6^ RNA copies/ml 21 days after treatment initiation. The disease severity according to the WHO ordinal clinical scale [17], any complications of COVID-19 and any drug-related adverse effects among groups were chosen as secondary endpoints.

Potential predictors of increased and/ or prolonged viral shedding were analysed: sex (male vs. female), age groups (< 65 years vs. ≥ 65 years), number of risk factors for severe COVID-19 according to the Centers for Disease Control and Prevention (< 5 vs. ≥ 5 risk factors) [18], immunodeficiency (yes vs. no), HM (yes vs. no), history of allogenic stem cell transplantation (yes vs. no), history of SOT (yes vs. no), vaccination status, treatment strategies (2 × DAAs ± 1 × mAB vs. 1 × DAA plus 1 × mAB), time of treatment initiation (≤ 5 days vs. > 5 days after diagnosis), WHO ordinal clinical severity scale score (1–3 vs. 4–7), and complicated course of COVID-19 (yes vs. no). Immunodeficiency was presumed in patients with a medical history of organ transplantation or autoimmune disease under immunosuppressive therapy, with underlying HM and/ or with advanced HIV-infection (CD4^+^ cell count < 200/µl). Patients who received the basic immunization and a first booster vaccination were considered fully vaccinated. Patients who had received less or no vaccinations were grouped as only partially vaccinated or not vaccinated, respectively.

Clinical data were anonymized and entered into an electronic case report form.

**Fig. 1:**
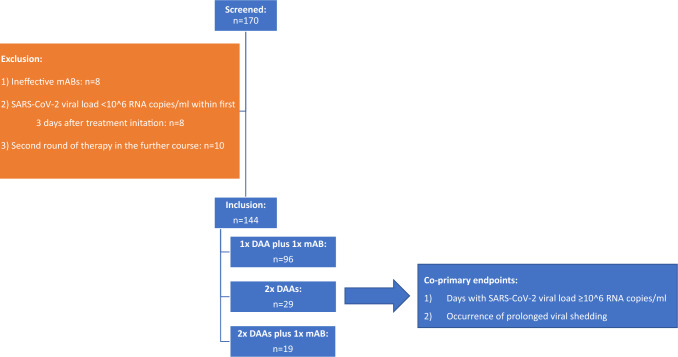
Subject disposition. 170 patients received primarily combined dual anti-SARS-CoV-2 therapies. Patients having received ineffective mABs or having highlighted a SARS-CoV-2 viral load < 10^6 RNA copies within the first three days were excluded. Furthermore, patients having received a second round of therapy due to clinical and/ or virological deterioration were excluded from the initial analysis. The latter were examined in a sub-analysis with regard to the effect of this second round of therapy on days with SARS-CoV-2 ≥ 10^6 RNA copies/ml Finally, 144 patients were included in the initial analysis having received 1 × DAA plus 1 × mAB, 2 × DAAs or 2 × DAAs plus 1 × mAB. *DAA* direct acting antiviral, *mAB* monoclonal antibody

Patients reaching a SARS-CoV-2 viral load < 10^6^ RNA copies/ml within the first three days after treatment initiation were excluded from the final analysis as the success of treatment could not be clearly attributed (*n* = 8). The same was applied when neutralizing capacity of mABs could not be assumed for a mismatch of virus variant versus mAB chosen (*n* = 8). Furthermore, cases with a second course of antiviral therapy due to lacking or insufficient clinical and/ or virological response were excluded (*n* = 10). Inclusion and exclusion criteria are summarized in Fig. 1.

Statistical analysis was carried out using R version 4.2.3 (R Core Team 2023: R: A Language and Environment for Statistical Computing, R Foundation for Statistical Computing, Vienna, Austria). Descriptive analyses included the calculation of medians and interquartile ranges for continuous variables and frequencies (absolute and relative) for categorical variables. Prolonged viral shedding was compared between treatment strategies and the patients’ characteristics mentioned above in univariable analyses using odds ratios and Fisher’s exact tests. Days with SARS-CoV-2 viral load $\ge$ 10^6^ RNA copies/ml were illustrated with Kaplan − Meier plots and compared between treatment strategies as well as patient characteristics using by *t* tests. Analogously, the days with SARS-CoV-2 viral load $\ge$ 10^6^ RNA copies/ml were analysed among patients who were excluded from the initial analysis, because they received a second round of antiviral therapy. Moreover, a sub-analysis on combinations of DAAs and mABs was performed using ANOVA. Finally, a multivariable linear regression analysis was carried out including age, sex and those predictors that were significant in univariable analyses. *P* values ≤ 0.05 were regarded as statistically significant.

## Results

### Study population

Of the 170 patients screened, who received first-line combined, dual anti-SARS-CoV-2 therapies, 144 were eligible for analysis (Fig. 1).

Immunodeficiency was present in 85.4% (*n* = 123/144), mostly due to immunosuppression following SOT (52.8%; *n* = 76/144) or underlying HM (28.5%; *n* = 41/144). In the 21 formally immunocompetent patients (14.6%), age > 65 years and > 2 comorbidities, representing ≥ 3 risk factors for severe COVID-19, were reasons for concern for a severe course of COVID-19. Virus genomic sequencing was conducted in 59.0% of patients (*n* = 85/144), with Omicron variants detected in 84 cases and a delta variant in only one. In all 85 patients, potential efficacy of the applied mAB could be assumed according to the virus variant [16]. Median treatment initiation was day 0 (range: 0–56; IQR 0–1) after first positive PCR test for SARS-CoV-2. Treatment categories included one DAA plus one mAB (66.7%; *n* = 96/144), two DAAs (20.1%; *n* = 29/144), or two DAAs plus one mAB (13.2%; *n* = 19/144). As there were no significant differences in viral shedding between the latter two groups (8.0 days [IQR 6.0–14.0] vs. 6.0 days [IQR 5.0–8.0]; *p* = 0.99), both groups were combined for further analyses (2 × DAAs ± 1 × mAB). Patient baseline characteristics are detailed in Table 1.

**Table 1:** Baseline characteristics and clinical parameters

Participants	144
Age category	
< 65 years [%]	65.3 (*n* = 81/144)
≥ 65 years [%]	43.7 (*n* = 63/144)
Sex category	
Male [%]	68.8 (*n* = 99/144)
Female [%]	31.2 (*n* = 45/144)
Risk factors for severe COVID-19	
Immunodeficiency*	85.4 (*n* = 123/144)
Solid organ transplantation with drug immunosuppression**	52.8 (*n* = 76/144)
Underlying haematological malignancies***	28.5 (*n* = 41/144)
Allogenic bone marrow transplantation with drug immunosuppression	6.9 (*n* = 10/144)
Chronic variable immunodeficiency	1.4 (*n* = 2/144)
HIV infection with CD4 + -cell count < 200/ µl	1.4 (*n* = 2/144)
Rheumatological diseases/ collagenoses/ vasculitides	3.5 (*n* = 5/144)
Solid cancer	9.0 (*n* = 13/144)
Chronic heart failure	9.7 (*n* = 14/144)
Coronary heart disease	12.5 (*n* = 18/144)
Atrial fibrillation	9.7 (*n* = 14/144)
Chronic liver disease	13.9 (*n* = 20/144)
Chronic kidney failure	12.5 (*n* = 18/144)
Dialysis	0.7 (*n* = 1/144)
Chronic pulmonary disease	13.9 (*n* = 20/144)
Diabetes mellitus	15.3 (*n* = 22/144)
Obesity	6.3 (*n* = 9/144)
Chronic inflammatory bowel disease	2.1 (*n* = 3/144)
Chronic neurologic diseases	9.7 (*n* = 14/144)
Cerebrovascular disease	2.1 (*n* = 3/144)
Chronic psychiatric diseases	1.4 (*n* = 2/144)
SARS-CoV-2 vaccination status	
Basic vaccination plus first booster vaccination [%]	72.9 (*n* = 105/144)
Not/partly vaccinated [%]	18.8 (*n* = 27/144)
Not documented [%]	8.3 (*n* = 12/144)
Variant of SARS-CoV-2	
Omicron [%]	58.3 (*n* = 84/144)
Delta [%]	0.7 (*n* = 1/144)
No sequencing [%]	41.0 (*n* = 59/144)
Therapeutical agents	
DAAs [absolute]	192
Remdesivir [%]	57.3 (*n* = 110/192)
Molnupiravir [%]	28.1 (*n* = 54/192)
Nirmatrelvir/ritonavir [%]	14.6 (*n* = 28/192)
mABs [absolute]	115
Sotrovimab [%]	74.8 (*n* = 86/115)
Tixagevimab/cilgavimab [%]	23.5 (*n* = 27/115)
Casirivimab/imdevimab [%]	1.7 (*n* = 2/115)
Days of treatment initiation after first positive PCR for SARS-CoV-2 [median]	0 (range: 0–56; IQR 0–1)
Combined therapy strategies	
1 × DAA plus 1 × mAB [%]	66.7 (*n* = 96/144)
Remdesivir + mAB [%]	66.7 (*n* = 64/96)
Nirmatrelvir/ritonavir + mAB [%]	19.8 (*n* = 19/96)
Molnupiravir + mAB [%]	13.5 (*n* = 13/96)
2 × DAAs [%]	20.1 (*n* = 29/144)
Remdesivir + molnupiravir [%]	79.3 (*n* = 23/29)
Remdesivir + nirmatrelvir/ritonavir [%]	17.3 (*n* = 5/29)
Molnupiravir + nirmatrelvir/ritonavir [%]	3.4 (*n* = 1/29)
2 × DAAs plus 1 × mAB [%]	13.2 (*n* = 19/144)
Remdesivir + molnupiravir + mAB [%]	84.2 (*n* = 16/19)
Remdesivir + nirmatrelvir/ritonavir + mAB [%]	10.5 (*n* = 2/19)
Molnupiravir + nirmatrelvir/ritonavir + mAB [%]	5.3 (*n* = 1/19)
WHO ordinal clinical severity scale [median score]	3 (range: 1–5; IQR: 3–3)
Complications of SARS-CoV-2 infection	
COVID-19 pneumonia [%]	13.2 (*n* = 19/144)
Oxygen therapy by nasal or mask prongs [%]	13.9 (*n* = 20/144)
Non-invasive ventilation or high-flow oxygen [%]	1.4 (*n* = 2/144)
Additional therapy with dexamethasone [%]	6.3 (*n* = 9/144)
Bacterial superinfection [%]	8.3 (*n* = 12/144)
Temporary elevation of amino transferases [%]	3.5 (*n* = 5/144)
Acute on chronic kidney failure [%]	2.1 (*n* = 3/144)
Temporary renal transplant deterioration [%]	1.4 (*n* = 2/144)
Hepatic encephalopathy [%]	0.7 (*n* = 1/144)
Delirium [%]	0.7 (*n* = 1/144)
Post COVID-19**** [%]	0.7 (*n* = 1/144)
Delay of oncological therapy [%]	12.2 (*n* = 5/41)
Possible treatment side effects	
Diarrhoea [%]	2.1 (*n* = 3/144)
Nausea and vomiting [%]	0.7 (*n* = 1/144)
SARS-CoV-2 RNA < 10^6 copies at day 21 after treatment initiation	
Yes [%]	85.4 (*n* = 123/144)
No [%]	14.6 (*n* = 21/144)
Days with SARS-CoV-2 RNA ≥ 10^6 copies	
All [median]	8.0 (IQR 6.0–15.3)
Under 1 × DAA plus 1 × mAB [median]	9.5 (IQR 6.0–14.0)
Under 2 × DAAs [median]	8.0 (IQR 6.0–14.0)
Under 2 × DAAs plus 1 × mAB [median]	6.0 (IQR 5.0–8.0)

*DAA* direct acting antiviral, *IQR* interquartile range, *mAB* monoclonal antibody

^*^Suspected in patients with a medical history of organ transplantation or autoimmune disease with concomitant immunosuppressive therapy, with underlying haematological malignancies and/ or with uncontrolled HIV-infection (CD4^+^ cell count < 200/µl)

^**^kidney transplantation (75·0%; *n* = 57/76), heart transplantation (15·8%; *n* = 12/76), liver transplantation (4·0%; *n* = 3/76), kidney and heart transplantation (2·6%; *n* = 2/76), kidney and pancreas transplantation (1·3%; *n* = 1/76), lung transplantation (1·3%; *n* = 1/76)

^***^Non-Hodgkin lymphoma (36**·**6%; *n* = 15/41), acute myeloid leukaemia (26**·**8%; *n* = 11/41), multiple myeloma (9**·**8%; *n* = 4/41), myeloproliferative neoplasms (9**·**8%; *n* = 4/41), myelodysplastic syndromes (7**·**3%; *n* = 3/41), acute lymphoblastic leukaemia (4**·**9; *n* = 2/41), undifferentiated leukaemia (2**·**4%; *p* = 1/41), aplastic anaemia (2**·**4%; *p* = 1/41)

^****^The rate might be higher as a follow-up time > 3 months was not given in all cases

### Days with SARS-CoV-2 viral load ≥ 10^6^ copies/ml

Median SARS-CoV-2 viral shedding ≥ 10^6^ copies/ml lasted for 8.0 days (IQR 6.0–15.3) following initiation of dual anti-SARS-CoV-2 treatment.

**Fig. 2:**
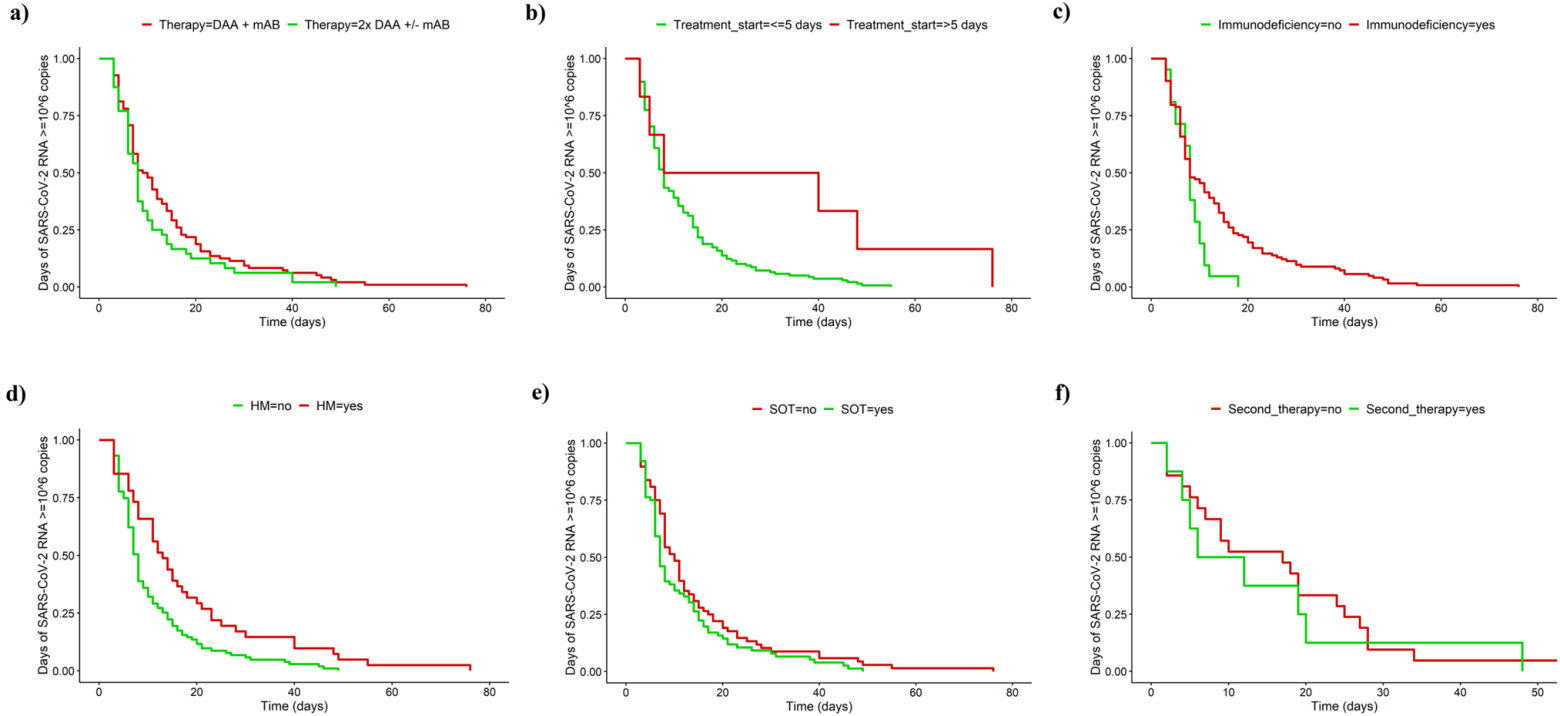
Viral Shedding. Kaplan–Meier plots showing the probability of SARS-CoV-2 RNA ≥ 10^6 copies/ml set in relation to time in days. The time of viral shedding was not significantly different between the treatment strategies (a: 1 × DAA + 1 × mAB: 14·0 days vs. 2 × DAA ± 1 × mAB: 11·0 days; *p* = 0·17). The period was longer in b) patients with a late treatment initiation (> 5 days after diagnosis: 30·7 days vs. ≤ 5 days after diagnosis: 12·3 days; *p* < 0·01), c) immunocompromised patients (13·9 days vs. 8·0 days in immunocompetent patients; *p* = 0·04) and/ or d) especially in patients with HM (17·1 days vs. 11·0 days in patients without HM; *p* < 0·01). In comparison, in patients under immunosuppressive medication following SOT the time was even shorter (11·7 days vs. 14·5 days in patients with no history of solid organ transplantation; *p* = 0·18). *DAA* direct acting antiviral, *HM* haematological malignancies, *mAB* monoclonal antibody, *SOT* solid organ transplantation

In univariable analysis, patients < 65 years had significantly longer viral shedding (10.4 vs. 15.0 days; *p* = 0.03). The time to viral load < 10^6^ copies/ml was significantly longer in patients with late treatment initiation (30.7 vs. 12.3 days; *p* < 0.01) and in immunocompromised patients (13.9 vs. 8.0 days; *p* = 0.04), especially in the subgroup of patients with underlying HM (18.1 vs. 11.0 days; *p* < 0.01). In contrast, patients receiving immunosuppressives post SOT more readily experienced a viral load < 10^6^ copies/ml (11.7 vs. 14.5 days; *p* = 0.17). Neither the different treatment strategies (2 × DAA ± 1 × mAB vs. 1 × DAA + 1 × mAB) nor the number of risk factors (< 5 vs. ≥ 5) resulted in statistically significant differences regarding that endpoint (Table 2). Kaplan − Meier plots were added to visualize the results (Fig. 2a–e).

**Table 2:** Factors influencing viral shedding (univariable analysis)

	Days with SARS-CoV-2 viral load ≥ 10^6 RNA copies/ml	95% CI	*p* value	Prolonged viral shedding	OR	95% CI	*p* value
Age category							
< 65 years	15.0	12.4–17.7	**0**·**03**	21.0 (*n* = 17/81)	3.9	1.2–16.8	**0**.**02**
≥ 65 years	10.4	7.5–13.4		6.3 (*n* = 4/63)			
Sex category							
Male	12.8	10.4–15.2	0.76	13.1 (*n* = 13/99)	0.7	0.2–2.1	0.46
Female	13.5	9.9–17.1		17.8 (*n* = 8/45)			
Number of risk factors for severe COVID-19							
< 5	13.5	11.4–15.6	0.14	15.9 (*n* = 21/132)	NA	NA	0.21
≥ 5	8.0	1.1–14.9		0 (*n* = 0/12)			
Immunodeficiency							
Yes	13.9	11.7–16.0	**0**.**04**	17.1 (*n* = 21/123)	NA	NA	**0**.**04**
No	8.0	2.9–13.2		0 (*n* = 0/21)			
Haematological malignancies							
Yes	18.1	14.5–21.7	** < 0**.**01**	26.8 (*n* = 11/41)	3.4	1.2–9.9	**0**.**02**
No	11.0	8.7–13.3		9.7 (*n* = 10/103)			
Allogenic bone marrow transplantation							
Yes	18.6	10.6–14.7	0·14	40.0 (*n* = 4/10)	4.5	0.9–21.4	**0**.**04**
No	12.6	11.0–26.2		12.7 (*n* = 17/134)			
Solid organ transplantation							
Yes	11.7	9.0–14·5	0.18	11.8 (*n* = 9/76)	0.6	0.2–1.8	0.35
No	14.5	11.6–17·4		17.6 (*n* = 12/68)			
Fully vaccinated (if documented)							
Yes	12.0	9.7–14.4	0.09	12.4 (*n* = 13/105)	0.5	0.2–1.8	0.22
No	16.6	12.0–21.3		22.2 (*n* = 6/27)			
Therapy strategies							
1 × DAA plus 1 × mAB	14.0	11.6–16.5	0.17	15.6 (*n* = 15/96)	1.3	0.4–4.4	0.80
2 × DAA ± 1 × mAB	11.0	7.6–14.5		12.5 (*n* = 6/48)			
Time of treatment initiation							
Early (≤ 5 days)	12.3	10.3–14.2	** < 0**.**01**	13.0 (*n* = 18/138)	0.2	< 0.1–1·2	**0**.**04**
Late (> 5 days)	30.7	21.3–40.0		500 (*n* = 3/6)			
Complicated course of COVID-19							
Yes	11.6	7.8–15.4	0·38	10.0 (*n* = 4/40)	0.6	0.1–1.9	0.43
No	13.6	11.2–15.9		16.3 (*n* = 17/104)			
WHO ordinal clinical severity scale							
1–3	13.2	11.0–15.4	0.67	14.8 (*n* = 18/122)	1.1	0.3–6.4	1.00
4–7	12.0	6.9–17.1		13.6 (*n* = 3/22)			

*DAA* direct acting antiviral, *mAB* monoclonal antibody, *NA* not applicable

Time with SARS-CoV-2 viral load ≥ 10^6 RNA copies/ml was significantly longer in patients aged < 65 years, patients under immunodeficiency, patients with underlying haematological malignancies and in cases with a late treatment initiation

Prolonged viral shedding (SARS-CoV-2 viral load ≥ 10^6 RNA copies/ml > 21 days) was significantly more frequently observed in patients aged < 65 years, immunocompromised patients, patients with underlying haematological malignancies, patients following a bone marrow transplantation and in cases with a late treatment initiation

**Fig. 3:**
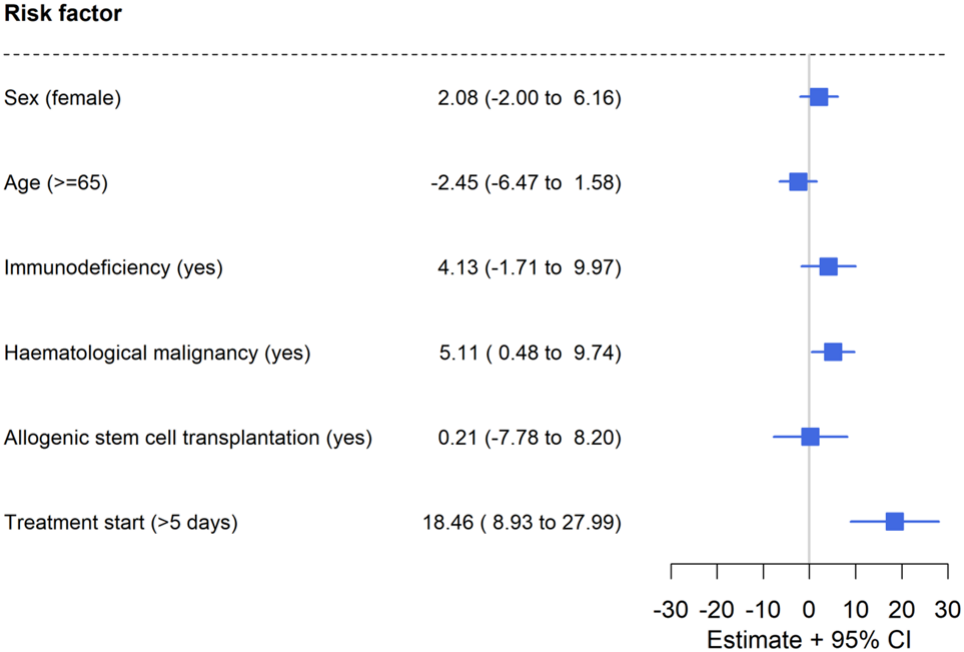
Factors influencing viral shedding (multivariable analysis). Forest plot visualizing the results of the multivariable analysis regarding factors being associated with longer viral shedding. Despite dual anti-SARS-CoV-2 therapy, viral shedding was significantly longer when treatment start was > 5 days after diagnosis of COVID-19 and/ or in patients with underlying haematological malignancies. The effects of sex, age, immunodeficiency, and/ or allogenic bone marrow transplantation were minor and insignificant

In multivariable analysis, underlying HM (*p* = 0.03) and treatment initiation > 5 days after diagnosis of COVID-19 (*p* < 0.01) were significantly associated with longer viral shedding (Fig. 3).

### Prolonged viral shedding

The second primary endpoint, i.e., achieve a SARS-CoV-2 viral load < 10^6^ copies/ml within 21 days, was achieved by 85.4% (*n* = 123/144) of patients (Table 1). Patients with HM had the highest rate of prolonged viral shedding (26.8%; *n* = 11/41; *p* = 0.02). In comparison, the risk for prolonged viral shedding was substantially lower in patients on immunosuppressive medication following SOT (11.8%; *n* = 9/76; *p* = 0.35).

Prolonged viral shedding was significantly more frequent in immunocompromised patients (*p* = 0.04) and in those with late treatment initiation (*p* = 0.04). Within the group of immunocompromised, patients with underlying HM (OR 3.5; 95% CI 1.2–9.9; *p* = 0.02) and patients on immunosuppressive medication following allogenic stem cell transplantation (OR 4.5; 95% CI 0.8–21.4; *p* = 0.04) were at highest risk for prolonged viral shedding. Of note, this led to a delay in cancer therapy in 12.2% (*n* = 5/41) of all patients with HM. In contrast, in recipients of immunosuppressives following SOT, dual anti-SARS-CoV-2 therapy was associated with lower rates of prolonged viral shedding (OR 0.6; 95% CI 0.2–1.8; *p* = 0.35). Patients < 65 years had a higher risk for prolonged viral shedding (OR 3.9; 95% CI 1.2–16.8; *p* = 0.02). Regarding the treatment strategies (2 × DAA ± 1 × mAB vs. 1 × DAA plus 1 × mAB), no significant difference was observed.

### Sub-analysis on combinations of DAAs and mABs

**Fig. 4:**
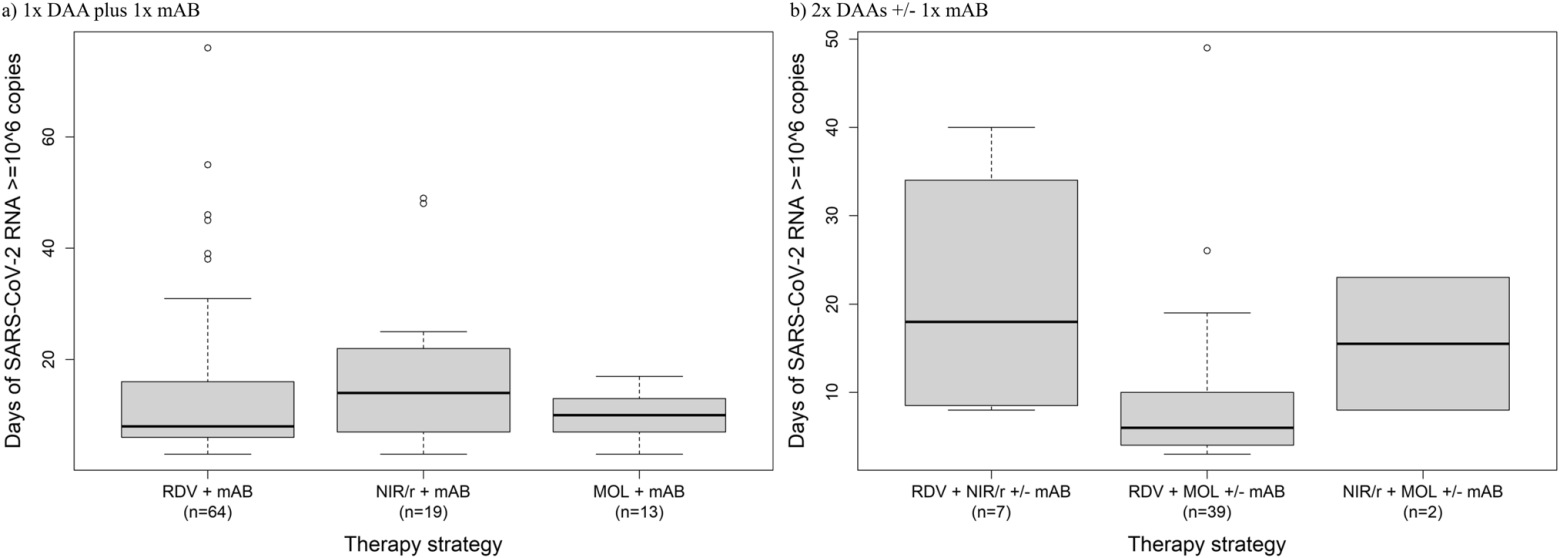
Sub-analysis on therapy strategies. Using an effective mAB, there was no difference in time with SARS-CoV-2 viral load ≥ 10^6 RNA copies/ml whether combining it with remdesivir (14.1 days), molnupiravir (9.9 days; *p* = 0.44) or nirmatrelvir/ritonavir (16.7 days; *p* = 0.30) (**a**). In contrast, the combination of remdesivir with molnupiravir appeared to be beneficial compared to the combination of remdesivir and nirmatrelvir/ritonavir (8.9 vs. 21.8 days; *p* < 0.01; **b**). Of note, nirmatrelvir/ritonavir could not be used in patients under immunosuppressive medication following organ transplantation due to potential drug-drug-interactions and was therefore rarely administered. The significance is thus weakened. *DAA* direct acting antiviral, *mAB* monoclonal antibody, *MOL* molnupiravir, *NIR/r* nirmatrelvir/ritonavir, *RDV* remdesivir

Regarding combinations with mABs, there was no significant difference in time with SARS-CoV-2 viral load ≥ 10^6^ RNA copies/ml depending on the DAA partner (Fig. 4a). In contrast, the combination of remdesivir with molnupiravir appeared to be beneficial as compared to the combination of remdesivir and nirmatrelvir/ritonavir (8.9 vs. 21.8 days; *p* < 0.01; Fig. 4b). Of note, nirmatrelvir/ritonavir was not used following SOT to avoid drug-drug interactions. As stated above, these patients had shorter viral shedding when compared with other subgroups of patients (Fig. 2e).

### Clinical course of COVID-19 and safety of dual anti-SARS-CoV-2 therapies

Patients had a median WHO ordinal clinical severity scale score of 3 (range: 1–5; IQR 3–3), i.e., having been hospitalized with no oxygen therapy (Table 1). A higher score (> 3) was not associated with longer viral shedding (Table 2). Most common complications were COVID-19 pneumonia (13.2%; *n* = 19/144) and bacterial superinfections (8.3%; *n* = 12/144). Most of the patients who required oxygen therapy were stable under oxygen by nasal or mask prongs (13.8%; *n* = 20/144), i.e., a WHO ordinal clinical severity scale score of 4. Only two patients (1.4%) required intensive care with high-flow oxygen (score of 5). No deaths were reported.

Diarrhoea and nausea with vomiting (2.1%; *n* = 3/144 and 0.7%; *n* = 1/144, respectively) were documented as potential adverse effects of dual anti-SARS-CoV-2 treatments. Temporary elevation of amino-transferases (2.1%; *n* = 3/144), acute on chronic kidney failure (1.4%; *n* = 2/144), and renal transplant functional deterioration (0.7%; *n* = 1/144) were considered as complications of COVID-19 rather than drug-induced.

### Impact of a second course of antiviral treatment

Among the 10 patients who were excluded from the analysis because they received a second round of antiviral therapy due to lack of clinical and/ or virological response, 8 patients fulfilled the criteria for prolonged viral shedding. To evaluate the potential impact of this second round of therapy, we compared the time to achieve a SARS-CoV-2 viral load < 10^6^ RNA copies/ml in these patients with the time in the 21 patients with prolonged viral shedding undergoing one course of dual therapy. The time was numerically reduced by 2.2 days in patients receiving a second round of therapy (*p* = 0.71) as shown in Fig. 1f.

## Discussion

In our cohort of 144 mainly immunocompromised patients receiving first-line combination therapies for COVID-19, clinical courses were mild to moderate and prolonged viral shedding was effectively prevented in 85.4% of these cases. Only two patients required intensive care and no deaths were documented. Our results are encouraging as they exceed those reported with monotherapy in vulnerable populations [6]. Clinical outcomes in patients infected with the SARS-CoV-2 Omicron variant are generally more favourable as compared to earlier virus variants, but still a proportion of 7.8% with treatment failure—defined as severe COVID-19 or COVID-19-related death—has been shown in patients with HM under monotherapy [19]. In our study, prolonged viral shedding was also observed primarily in patients with HM despite dual therapies (14.6%; *n* = 11/144), which once again reveals this group as particularly vulnerable.

SOT patients are also prone to prolonged viral shedding [20]. In our study, persistent viral shedding was less common in this group of patients. The benefit of combined antiviral therapy in these patients appeared to be less pronounced than in patients with HM.

Interestingly, the total number of risk factors for severe COVID-19 had no effect on viral shedding, suggesting that the underlying disease itself has a greater impact than the number of different conditions. Moreover, no association between the clinical course and viral shedding was found.

While the Omicron sub-lineages are associated with milder COVID-19 courses, yet a significant proportion of immunocompromised and older patients are still at risk for severe COVID-19 including death [21].

Prolonged viral shedding delayed antineoplastic treatment in 12.2% of the patients included in this study. This is in line with a study in patients with simultaneous diagnosis of HM and COVID-19 observing frequent (17.2%) substantial delays resulting in significantly higher 30-day mortality [8].

The benefit of DAA in combination with a mAB found in our cohort is consistent with other case reports and small series [14, 22].

However, escape mutations have been observed during mAB exposure, and viral evolution has eventually made all available mABs ineffective, while DAAs retained efficacy [7, 23]. Therefore, treatment combinations including two DAAs seem to be promising and have already shown favourable results in a small case series with patients suffering mostly from HM and/ or receiving anti-CD20 treatment [15]. The case series reports a 30-day virological and clinical response of 73% and a low rate of adverse side effects. In some cases of failure of combined treatment, a repeated course with longer duration was successful. Moreover, the addition of mABs in 18 out of 22 patients was associated with improved therapeutic response. In a recently presented (yet not published as a journal article) study, four of 15 patients with underlying HM experienced a relapse or re-infection despite a five-day course of nirmatrelvir/ritonavir plus remdesivir. In six patients with additional tixagevimab/cilgavimab, no relapse or re-infection was observed [24]. Other than in these studies, we did not see a significant impact of a triple therapy with an additional mAB (*p* = 0.99) (Table 1).

According to the pivotal studies, nirmatrelvir/ritonavir and remdesivir as single compounds had higher efficacy than molnupiravir [2–4]. Therefore, it is conceivable, that combination therapies consisting of nirmatrelvir/ritonavir and remdesivir may be more effective than combinations of any of the two compounds with molnupiravir. The use of nirmatrelvir/ritonavir, however, is limited due to a large number of drug-drug interactions [25]. This applies especially for patients after SOT who frequently use calcineurin-inhibitors, such as tacrolimus, to prevent graft rejection. Co-administration with ritonavir may result in a severe increase in tacrolimus serum levels [26]. Thus, in our study with a high proportion of SOT patients (*n* = 76/144, 52.8%), the combination of remdesivir and molnupiravir was most frequently chosen (*n* = 39, additional mAB in 16 cases)—especially in SOT patients. The second most frequent combination was nirmatrelvir/ritonavir and remdesivir (*n* = 7), including two cases with additional mAB, followed by nirmatrelvir/ritonavir plus molnupiravir (*n* = 2, including one case with additional mAB). Therefore, the observed benefit of remdesivir combined with molnupiravir as compared to remdesivir and nirmatrelvir/ritonavir most likely reflects a difference in the groups treated. The same is true for the finding that younger patients were more likely to have prolonged viral shedding: treatment in this group is often more aggressive and immunosuppressive.

In summary, none of the different treatment strategies (1 × DAA + 1 × mAB vs. 2 × DAA ± 1 × mAB) proved to be superior, while an early treatment initiation was proofed to be favourable.

According to the previously described study and the case reports, in some cases of treatment failure, repeated or extended courses of treatment were ultimately successful in stopping viral shedding [11–13]. In eight patients out of our subgroup of 11 patients with repeated therapies due to initial treatment failure, viral shedding was still observed 21 days after initial treatment initiation, and the duration of viral shedding was not significantly reduced. Nevertheless, viral clearance was ultimately achieved in all patients enrolled.

The low number of adverse effects attributed to the drugs used in this study suggests good tolerability and safety of the combinations used. However, it should be noted that other reports described cardiac events (one myocardial infarction, one bradycardia) that may be related to treatment [15]. In our study, no treatment was discontinued because of adverse effects.

Some of the patients treated in our departments presented with impaired kidney function at baseline. Renal function rarely deteriorated during dual treatment. This was even true in treatment initiated at glomerular filtration rates below 30 ml/min*1.73 m^2^. In the meantime data indicate that above all remdesivir can be used in reduced kidney function [27].

Limitations of our study are inherent to its retrospective design. Treatment algorithms differed between the four participating centres, reflecting the lack of knowledge at the time. Our patient group is heterogeneous by baseline factors including comorbidities and causes of immunodeficiency. In some SOT patients, medical immunosuppression was temporarily reduced to improve immune response to SARS-CoV-2 infection. A potential effect could not be discriminated from the effect of antiviral therapy.

A control group is missing. This is mainly due to the fact that at a certain point in time, high-risk patients were regularly treated with combination therapies at the centres. Depriving some patients of this option did not seem to be ethically justifiable. In particular, because of other prevalent virus variants at other times, it was decided against recording a retrospective control group with an assumed bias that was too large.

Due to the defined primary endpoints of time to virus elimination and prolonged viral shedding, patients who had COVID-19 but died soon after diagnosis for any reason and/or did not receive any antiviral therapy were not included in this study, displaying a certain selection bias. The same applies for patients who did not respond to monotherapy and were therefore secondarily treated with combined antiviral regimens. Since the mABs in our study lost efficacy due to viral evolution, treatment combinations involving mABs can currently not be relied on.

In recipients of drugs interacting with ritonavir metabolism pathway, nirmatrelvir use may be limited. The use of nirmatrelvir/ritonavir in these patients would require a high level of expertise in complex interaction management, including close monitoring of immunosuppressant levels. Molnupiravir is no longer available in the European Union, but remains a treatment option in other world regions [28]. Overall, the suitable drug combinations are thus less numerous than at the time of our study, leaving a highlight on the combination of remdesivir with nirmatrelvir/ritonavir.

In conclusion, patients with HM benefited most from early dual anti-SARS-CoV-2 treatment. Combination therapy may also be beneficial in other immunocompromised patients as toxicity was low and viral clearance rates were high. For the time being, cautious individualized approaches should consider dual therapy.

Immunocompromised carriers of SARS-CoV-2 are considered a major factor in viral evolution as well as in maintaining epidemic outbreaks, especially under insufficient treatment [29]. Additionally to the individual benefits in these vulnerable patient group by reducing the risk of progression to severe COVID-19, by enabling participation in social life, and by allowing timely application of scheduled therapies, combined antiviral therapies may thus have additional positive effects on public health by decelerating viral evolution.

## Data Availability

The datasets analysed in this study are available for research purposes on reasonable request from the corresponding author. This applies for a maximum of ten years and only includes anonymised data whose disclosure does not interfere with the patients' interest.
